# Arrhythmia Classification with Single-Channel Features Extracted from “A Large-Scale 12-Lead ECG Database for Arrhythmia Study”

**DOI:** 10.3390/s25185621

**Published:** 2025-09-09

**Authors:** Monica Fira, Liviu Goraș, Lucian Fira, Radu Florin Popa, Hariton-Nicolae Costin

**Affiliations:** 1 Institute of Computer Science, Romanian Academy, Iasi Branch, 700481 Iasi, Romania; lgoras@etti.tuiasi.ro (L.G.); hariton.costin@iit.academiaromana-is.ro (H.-N.C.); 2 Faculty of Electronics, Telecommunications & Information Technology, Gheorghe Asachi Technical University of Iasi, 700050 Iasi, Romania; lfira@etti.tuiasi.ro; 3 Department of Vascular Surgery, Grigore T. Popa University of Medicine and Pharmacy of Iasi, 700115 Iasi, Romania; radu.popa@umfiasi.ro

**Keywords:** ECG, arrhythmia classification, feature extraction, machine learning

## Abstract

This study assesses how classical and modern features extracted from a single ECG lead (II) influence automated arrhythmia classification. Using the *Large Scale 12-Lead Electrocardiogram Database for Arrhythmia Study* and MATLAB^®^, we compared traditional morphological measures (e.g., QRS duration, QT interval, atrial/ventricular rates) with advanced time-, frequency-, and nonlinear-domain descriptors. The method classifies ECGs into four or eight categories using 15–39 features, either automatically selected or combined. In the eight-class task, 29–39 features yielded 69% accuracy; in the four-class task, 15 MRMR-selected features achieved 94.2% accuracy. A key strength is efficiency: relying on a single lead reduces preprocessing, storage, and classification time by a factor of ~12 compared with 12-lead approaches. These findings show that advanced descriptors from a single lead can match multi-lead performance, enabling practical, scalable clinical applications.

## 1. Introduction

The electrocardiogram (ECG) represents one of the most widely employed non-invasive techniques for assessing the electrical activity of the heart and holds a central role in the diagnosis of a broad spectrum of cardiovascular diseases, ranging from arrhythmias to myocardial ischemia. In clinical settings, ECG interpretation traditionally relies on a standardized set of morphological and temporal features—such as QRS complex duration, QT interval, R and T wave axes, and ventricular rate—which are highly interpretable and well established in medical practice. Nonetheless, these conventional features may prove insufficient in capturing the full complexity and subtle variations of cardiac dynamics, particularly in early-stage or atypical pathological conditions, as well as in automated diagnostic contexts.

The integration of advanced signal processing methods and machine learning techniques has facilitated the extraction of a more comprehensive and informative set of features from ECG signals. These include not only traditional time-domain and frequency-domain descriptors, but also nonlinear and statistical measures capable of characterizing the signal complexity and variability. Features such as heart rate variability (HRV), spectral energy distribution, and entropy have demonstrated their utility in complementing classical parameters, thereby enhancing the sensitivity and specificity of automated classification systems for cardiac pathology.

Class separability estimation plays a fundamental role in the preliminary analysis of a dataset potential for classification, influencing feature selection, the design of learning algorithm architectures, and the assessment of problem complexity. Separability refers to the degree to which instances belonging to different classes are distinctly distributed within the feature space. Its estimation can be approached using a combination of statistical, geometric, informational, and visual methods.

Numerical techniques include clustering and dispersion metrics, such as the Silhouette score [1], which evaluates the difference between intra- and inter-cluster distances for each sample; the Davies–Bouldin Index [2], which quantifies the ratio between intra-cluster dispersion and inter-cluster separation; and the Calinski–Harabasz Index [3], which reflects the ratio between global variance and within-cluster variance. Other relevant measures include the Dunn Index [3,4], which seeks to maximize the minimum inter-cluster distance while minimizing intra-cluster dispersion, and the Shannon–Wiener entropy index computed over local label neighborhoods, particularly informative in manifold-structured data.

Additionally, theoretical estimates of class overlap can be derived from distance-based measures such as Bhattacharyya, Mahalanobis, and Jeffries–Matusita distances, which characterize the divergence between probabilistic class distributions. From the perspective of feature interrelations, tools such as correlation matrices, mutual information, and eigenvalue analysis of the covariance matrix can reveal redundancy, collinearity, and opportunities for efficient dimensionality reduction [5,6].

For more intuitive interpretation, two-dimensional projection techniques are frequently employed. These include Principal Component Analysis (PCA)—which linearly preserves global variance—and nonlinear methods such as t-distributed Stochastic Neighbor Embedding (t-SNE), Uniform Manifold Approximation and Projection (UMAP), and Isometric Mapping (Isomap), which preserve local neighborhood structures and can uncover latent clusters in the data [7]. Moreover, separability can be empirically evaluated by training simple classifiers (e.g., k-Nearest Neighbors with k = 1 or 3, Linear Discriminant Analysis, or linear Support Vector Machines) without parameter tuning, as a proxy for the intrinsic discriminative capacity of the feature set [8].

The integration of these complementary techniques offers a multi-perspective understanding of the internal structure of the dataset and supports a well-founded design of the subsequent stages in the machine learning pipeline [9].

The aim of this study is to analyze and compare different types of features extracted from ECG signals, with respect to their utility in automated classification tasks. Specifically, we investigate the performance of traditional features—such as QRS duration, QT interval, ventricular rate, atrial rate, R axis, T axis, Q onset, Q offset, and T offset—in relation to an extended set of modern features derived from the time domain, frequency (spectral) domain, and nonlinear analysis of the signal [9,10]. This comparison seeks to highlight the substantive potential of advanced features in enhancing computer-aided diagnostic systems, particularly within the frameworks of big data and machine learning. By integrating these complementary perspectives, the present study contributes to a more comprehensive understanding of how temporal, spectral, and ECG signal complexity features can be effectively combined with classical morphological characteristics—such as wave shape and onset/offset timings—to improve automated classification, facilitate early pathology detection, and support clinical decision-making.

The results show that the use of a limited and carefully selected set of general and signal-specific features, extracted solely from lead II, can produce ECG classification accuracies comparable to methods utilizing hundreds of features from all 12 leads. Thus, the method offers significant advantages in reducing computational costs, preprocessing time, and implementation complexity, making it well-suited for practical clinical applications and resource-constrained automated diagnostic systems.

## 2. Background

Ref. [11] describes an extensive 12-lead ECG database collected from over 10,000 patients, primarily used in arrhythmia research. The features employed include both temporal characteristics (such as QRS duration and RR interval) and morphological attributes (including the amplitudes of the P, R, and T waves), aimed at enabling the development of robust models for arrhythmia detection. The authors evaluated this database using a deep convolutional neural network (CNN). The obtained results indicate a maximum F1 score of 0.97, demonstrating the utility of the database both for training complex models and for validating their performance in realistic clinical settings. Additionally, when grouping the labels into four primary cardiac rhythm classes—sinus rhythm, atrial fibrillation, supraventricular tachycardia, and sinus bradycardia—the model achieved an average F1 score of 0.97. These results were reported using an 80:20 train–test data split. The findings underscore the potential of the PTB-XL database not only for multi-label learning but also for simplified classifications that are clinically relevant. The database is recognized as one of the most representative public resources for machine learning applications in electrocardiography.

In study [12], the PTB-XL database is presented in detail, comprising 12-lead ECG recordings with more than 21,000 samples from 18,000 patients. The authors extracted both raw features (complete waveform shapes) and clinical metadata (diagnostic labels, heart rate, electrical axis, etc.) to support the development and validation of automatic ECG signal classification algorithms. Within this study, the authors evaluated the performance of various deep learning models, including one-dimensional convolutional neural networks (1D CNNs) and sequential architectures such as ResNet and Inception. These models achieved macro F1 scores of up to 0.43 for the automated classification of five proposed diagnostic classes, consisting of combinations of standard cardiac labels. The results highlight the complexity of multi-label classification tasks in the ECG domain, while also demonstrating the high potential of this database as a benchmark resource for future research in the field.

Study [13] proposes a three-step process consisting of noise reduction (using band-pass filtering, LOESS, and Non-Local Means), feature extraction based on percentiles of interval duration and amplitude ratios, and classification using Extreme Gradient Boosting (XGBoost). The model was trained on a proprietary large dataset comprising 40,258 12-lead ECG recordings labeled across four rhythm classes: atrial fibrillation, supraventricular tachycardia, sinus bradycardia, and (possibly irregular) sinus rhythm. For patients without associated cardiac pathologies, the model achieved an F1 score of 0.988, while for those with additional cardiac conditions, the F1 score was 0.97. External validation using the MIT BIH Arrhythmia Database yielded an average F1 score of 0.992 via 10-fold cross-validation. These results position the method as comparable to cardiologist-level performance and demonstrate robust compatibility across different ECG signal sources.

In ref. [14], a classification method for arrhythmias is proposed based on a blending ensemble model, wherein multiple algorithms (Random Forest, SVM, XGBoost, etc.) are combined through a meta-model using Logistic Regression. The features employed include ECG intervals (PR, QT, ST), wave amplitudes, and heart rate variability statistics (mean and standard deviation of RR intervals), extracted using NeuroKit2 and automatically selected. The evaluation was conducted on the Chapman ECG database, which comprises 12-lead recordings from more than 10,000 patients. The proposed model achieved an accuracy of 96.48% with an 80:20 train–test split, outperforming individual methods and demonstrating the efficacy of ensemble strategies in ECG signal classification.

It is important to note that, generally, all reported results on these databases involve a 20% data split for testing; however, they often do not specify performance metrics obtained when the test set contains a balanced number of examples from each class. In the case of multi-class classification, the selection of the test set can significantly bias the results toward false positives if the data distribution is favorable. Specifically, if one class is easily separable and dominant in both training and test sets, a high classification accuracy does not necessarily imply correct classification of samples from other classes. Rather, the model may primarily learn to identify the majority class well, potentially inflating the overall performance metrics without adequately addressing minority classes.

Building on the aforementioned considerations regarding testing protocols, this study presents a highly rigorous evaluation methodology. The reported results are slightly lower—by a few percentage points—compared to those found in the literature for this database; however, they are obtained using the most stringent possible procedures. In a multi-class database with uneven class distribution, differences of a few percentage points in classification accuracy can often be attributed to the method of test set selection. Specifically, it is possible to artificially inflate accuracy by selecting the test set as a fixed percentage of the entire dataset, particularly if some classes are easily distinguishable and dominate in terms of sample count. This contrasts with a more robust approach in which the test set is randomly selected with balanced numbers of examples per class.

## 3. Materials and Methods

### 3.1. The ECG Database Used

The simulations and analyses presented in this study were conducted using an extensive collection of 12-lead electrocardiograms (ECG) obtained from over 10,000 patients. This database was specifically designed to support research in the automatic recognition of arrhythmias and represents one of the most comprehensive publicly available resources for this purpose [15]. The data were collected under real clinical conditions from patients with diverse demographic and pathological profiles, providing the database with significant variability and practical relevance for the development of robust algorithmic models.

Each recording includes synchronized ECG signals from the 12 standard leads. The duration of the signals varies but is generally sufficient to capture relevant cardiac events, typically lasting around 10 s. The sampling frequency is 500 Hz, allowing preservation of important cardiac signal details and being adequate for high-fidelity spectral and temporal analysis applications. The recordings are accompanied by clinical labels indicating the type of arrhythmia identified, if present, or confirming normal sinus rhythm. Additionally, some recordings include supplementary demographic information such as patient age and sex.

The database is available through the PhysioNet under the title “A 12-lead electrocardiogram database for arrhythmia research covering more than 10,000 patients” [15,16].

Among the main advantages of this database are its considerable size, which enables the development of machine learning algorithms with high generalization capacity, and the wide variety of cardiac conditions represented. The types of arrhythmias covered include, among others, atrial fibrillation, atrial flutter, ventricular tachycardias, and various forms of atrioventricular block. Furthermore, the signal annotation was performed by specialized medical personnel, contributing to the accuracy and credibility of the diagnosis associated with each recording.

However, this database also presents certain limitations. Among these is the imbalance in the distribution of arrhythmia classes, which may adversely affect the performance of classification algorithms, particularly in recognizing rare pathologies. Additionally, not all recordings include detailed clinical information about the patients, and in some cases, the signals may have already undergone preprocessing, limiting control over the initial stages of analysis.

This database has been widely used in the scientific literature and serves as a reference point in numerous studies focused on ECG signal classification [11,13]. Furthermore, it has been included in major international competitions, such as those organized by the PhysioNet/Computing in Cardiology Challenge [17], thereby contributing to the standardization and comparability of results within the scientific community. Due to its high quality and extensive coverage, this resource is particularly well-suited for testing advanced arrhythmia recognition algorithms, including those based on neural networks, deep learning methods, or hybrid classifiers. Furthermore, in the description of this database, the authors of ref. [11] highlight it as containing the largest number of subjects, the highest sampling rate, and the greatest number of leads—namely, 12 ECG channels. This ECG signal database encompasses 11 cardiac rhythms and 56 types of cardiovascular conditions, all annotated by professional physicians. Additionally, the database includes basic ECG measurements such as the number of QRS complexes, atrial and ventricular beat rates, Q offset, and T offset.

**Figure 1:**
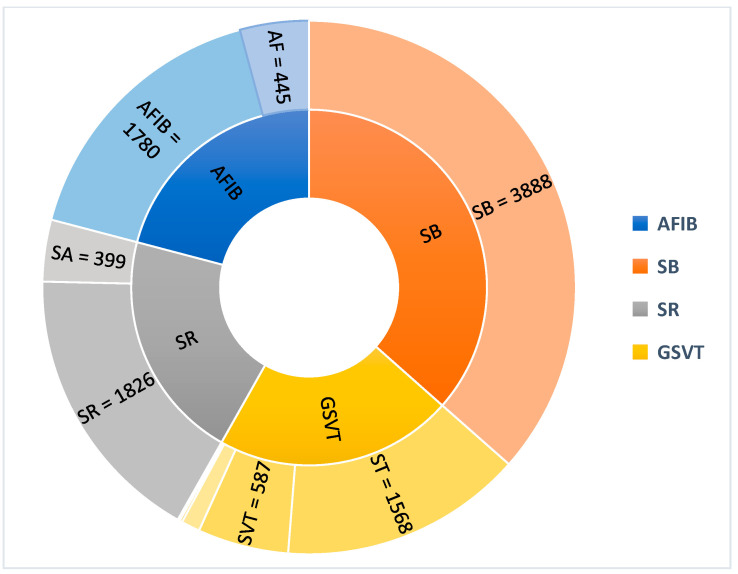
The distribution of data into the 11 classes, respectively, and their division into 4 main heart rate classes (AFIB = Atrial Fibrillation; SB = Sinus Bradycardia; SR = Sinus Rhythm; GSVT = General Supraventricular Tachycardia).

Figure 1 illustrates the distribution of the number of examples per cardiac rhythm class (outer ring) and their grouping into broader general rhythm categories. This grouping follows the scheme presented in ref. [11], which is motivated both by the clinical classification of cardiac pathologies into major categories and by the statistical considerations of the dataset. From the perspective of data usage for training, testing, and reporting results, it is mathematically and statistically challenging—if not impossible—to effectively utilize classes containing fewer than 10 examples. In both medical and engineering fields, it is commonly accepted that statistical analysis is unreliable for sample sizes smaller than 10. Consequently, classes such as SAAWR (7 examples) and AVRT (8 examples), as well as AVNRT (16 examples), were excluded from further analysis due to their insufficient representation. This rationale underlies the reporting of classification results using both eight classes and four classes in this work. The eight-class results exclude the underrepresented three classes mentioned above. Additionally, to provide a more representative evaluation, a four-class classification was performed by merging subclasses into broader rhythm categories.

### 3.2. Data Organization

The portion of the database used in this study (10,646 patients) consists of four components: raw ECG data, denoised ECG data, a diagnostic file, and an attribute dictionary file. For each subject, the raw ECG signals are stored as individual CSV files, while the corresponding denoised ECG signals are saved under the same filename in a separate folder. Each of these CSV files contains 5000 rows and 12 columns, with header names indicating the ECG leads. From these 12 leads, we used only the column corresponding to lead II (DII). All CSV files are uniquely identified by an ID, which is also referenced in the diagnostic file under the attribute name “FileName.” The diagnostic file includes all diagnostic information for each subject, such as the filename, rhythm type, comorbid conditions, patient age, sex, and other ECG summary attributes (obtained from the GE MUSE system).

We extracted both the classical ECG waveform features [11] and additional features proposed in this study, including time-domain features, spectral characteristics, and nonlinear signal measures. Based on these, we constructed a dataset consisting of 38 columns and 10,646 rows, where each row corresponds to a patient and each column represents a feature extracted from the ECG signal associated with lead II (DII). This dataset was further augmented with a 39th column representing the cardiac rhythm label, which was retrieved from the diagnostic file of the original database.

### 3.3. Analysis of Data Separability for 11-Class and 8-Class Classification

**Figure 2:**
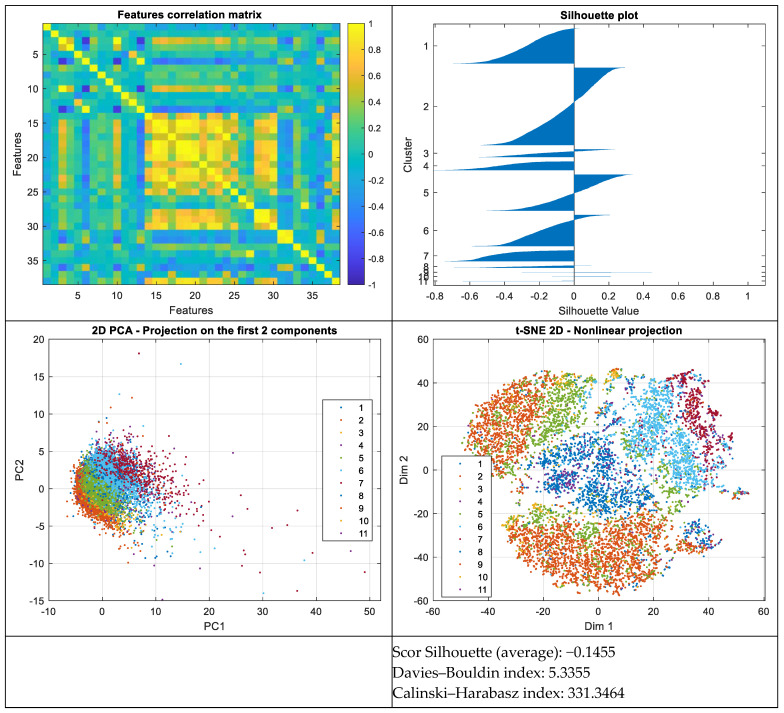
Data distribution for the 11 classes and separability measures.

To rigorously assess the ability of the extracted ECG signal features to support effective classification into the 11 predefined classes, a comprehensive analysis was conducted on the distribution of observations within the feature space. For this purpose, several standard multiclass separability metrics were employed, including the Silhouette score, the Davies–Bouldin Index (DBI), and the Calinski–Harabasz Index (CHI). Additionally, dimensionality reduction techniques such as Principal Component Analysis (PCA) and t-distributed Stochastic Neighbor Embedding (t-SNE) were applied to enable visualization of class distributions in two-dimensional projections (see Figure 2).

In clustering analysis and dimensionality reduction, assessing the quality of group separation and representing data in lower-dimensional spaces are key steps. The Silhouette score measures how well each data point fits within its assigned cluster compared to other clusters, with values close to 1 indicating clear separation. The Davies–Bouldin Index (DBI) quantifies the similarity between clusters, where lower values suggest better separation and higher compactness. The Calinski–Harabasz Index (CHI) evaluates the ratio between the variance between clusters and the variance within clusters, with higher values indicating well-defined structures. For visualization and complexity reduction, Principal Component Analysis (PCA) projects the data onto a set of orthogonal axes that capture maximum variance, while t-distributed Stochastic Neighbor Embedding (t-SNE) creates two- or three-dimensional representations that preserve local structures and neighborhood relationships, making it particularly useful for complex and nonlinear data.

The average Silhouette score, computed based on the ground-truth labels, yielded a value of −0.1455, which is substantially more negative than in the scenarios with fewer classes (as discussed in the following section). This negative score suggests that, on average, the instances are located closer to neighboring clusters than to their own, indicating poor intra-class cohesion and significant overlap between classes.

The Davies–Bouldin Index (DBI), with a value of 5.3355, is considerably elevated, reflecting high intra-class dispersion and low inter-class separation. Such a high DBI value implies poor separability between clusters in the feature space, which may adversely affect the performance of classification algorithms.

The Calinski–Harabasz Index (CHI) registered a value of 331.3464, which is noticeably lower than that obtained for the classification into four classes. This relatively low score indicates that the data does not form compact and well-separated groups, suggesting that the division into 11 classes may represent an artificial over-partitioning of a feature space lacking an inherent multi-class structure.

To further examine the internal geometry of the data, two-dimensional projection techniques were employed. Principal Component Analysis (PCA), a linear dimensionality reduction method, was applied to capture the directions of maximum variance. The projection onto the first two principal components did not reveal any clear cluster formation, but rather a diffuse distribution with no obvious organization, reinforcing the difficulty of achieving separation along the primary axes of variance.

In parallel, t-distributed Stochastic Neighbor Embedding (t-SNE), a nonlinear technique sensitive to local structure, was applied for two-dimensional visualization. The resulting t-SNE projection also failed to demonstrate any meaningful class segregation, revealing instead extensive mixing between classes. This indicates a complex latent topology and the absence of a structure naturally separable into 11 distinct clusters.

Furthermore, the feature correlation matrix revealed strong correlations among several pairs of variables, indicating the presence of informational redundancy. Such redundancy can impair the ability of classification models to extract meaningful patterns and supports the need for feature selection or transformation techniques (e.g., PCA or Linear Discriminant Analysis—LDA) during the preprocessing phase.

Overall, these findings suggest that, in its current form, the distribution of instances in the feature space does not support robust classification into 11 classes. Classifier performance may be improved by reducing dimensionality, removing redundant features, or generating more discriminative feature representations.

Based on these observations, the average scores for the Silhouette, Davies–Bouldin, and Calinski–Harabasz indices were recalculated considering only the classes that are well and moderately represented in terms of sample size, by excluding classes with fewer than 20 samples (specifically AVNRT, SAAWR, and AVRT). The resulting values for the eight-class classification scenario are as follows:

- Average Silhouette score: −0.0338
- Davies–Bouldin index: 4.6100
- Calinski–Harabasz index: 466.7599

### 3.4. Analysis of Data Separability for Four-Class Classification

The Silhouette plot exhibits both positive and negative values, indicating weak separability between classes. A considerable number of instances have negative scores, implying that they are closer to neighboring classes than to their assigned class, which reflects significant overlap among groups. Only a portion of the points show moderately positive scores, while the majority lie near zero, suggesting a poorly defined class structure within the current feature space.

The analysis of clustering evaluation methods indicates weak separability of the data into four classes, as evidenced by a negative mean Silhouette score (−0.0217), a relatively high Davies–Bouldin index (3.2165), and a moderate Calinski–Harabasz value (856.1679). These results suggest that clearly delineating classes within the current feature space is challenging, and achieving high classification accuracy is unlikely without further feature transformation or selection.

**Figure 3:**
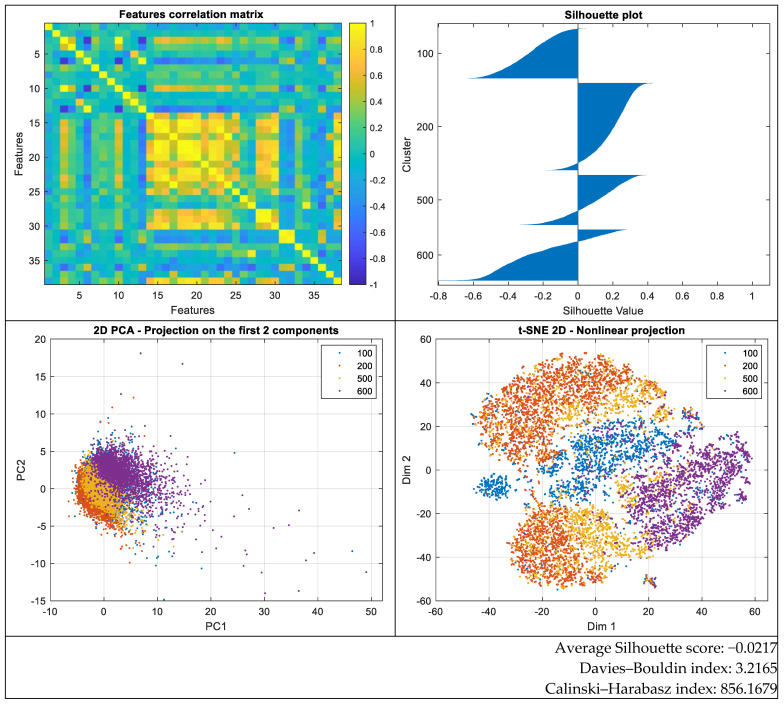
Data distribution for the four classes and separability measures.

Comparing the results obtained for the 11-class and 4-class scenarios reveals significant differences in the internal structure of the data. Firstly, the mean Silhouette score was negative in both cases (−0.0338 for eight classes and −0.0217 for four classes), indicating overlap between groups and low-class cohesion. However, the less negative value in the four-class classification (see Figure 3) suggests a slight improvement in intra-group coherence and inter-class separation.

The Davies–Bouldin index, which measures the ratio of within-cluster scatter to between-cluster separation, decreased from 4.6100 for eight classes to 3.2165 for four classes. Since lower values of this index reflect clearer cluster separation, this result implies that the features are significantly better separated when the number of classes is reduced.

Similarly, the Calinski–Harabasz index, quantifying the ratio of between-cluster variance to within-cluster variance, showed a substantial increase in the four-class scenario (from 466.76 to 856.17). This behavior is characteristic of a feature space where groups are more compact and well-defined.

Overall, the analysis of these three metrics highlights that the feature space is better structured and allows for clearer class separation in the four-class classification compared to the eight-class variant. This improvement can be explained by a reduced overlap between the fewer classes and a lower complexity of the classification problem. Therefore, from the perspective of data separability, reducing the number of classes leads to a marked enhancement in cluster structure and potentially contributes to superior classifier performance.

### 3.5. Features Used

In this study, two types of features were used, namely

(i) Features related to the ECG waveform presented in ref. [11];
(ii) Features in the time domain, frequency, and signal-complexity measures [10].

Both types of features were extracted from the DII lead. Since the presence of noise can pose a significant obstacle to any statistical analysis, we opted for a methodology in which the first step was noise removal.

In general, the sources of noise contamination in the ECG data from this database were due to power line interference, electrode contact noise, motion artefacts, muscle contractions, baseline wander, and random noise. Therefore, to eliminate noise, we applied a band-pass filter that retained only the frequency range between 0.5 Hz and 50 Hz. This was followed by LOESS (Locally Weighted Scatterplot Smoothing) smoothing to remove baseline wander effects and subsequently by Non-Local Means (NLM), an advanced denoising method. Unlike classical filtering techniques that consider only neighboring points, Non-Local Means searches for similar patterns throughout the entire signal, not just in the vicinity. The application of this technique results in a smoothed version of the signal that preserves genuine details while eliminating noise. Thus, this method retains structural information and reduces noise without blurring signal features.

The denoising technique used is the one presented in ref. [11]. We chose to maintain the same denoising method to ensure a fair comparison of the results and because it represents a sound methodological approach.

#### 3.5.1. Morphological Features of the ECG Waveform

The features extracted from lead II include ref. [11]:

- Ventricular rate in BPM (beats per minute),
- Atrial rate in BPM
- QRS duration in msec
- QT interval in msec
- Corrected QT interval in msec,
- R axis
- T axis
- QRS count
- Q onset (samples)
- Q offset (samples)
- T offset (samples)

#### 3.5.2. Features in the Time Domain, Frequency and Signal-Complexity Measures

Information regarding age and sex was retained as features, given its relevance in virtually all medical data analyses.

Based on the preprocessed lead II signals, we extracted features from the time and frequency domains, along with nonlinear dynamics measures. These features were also utilized in ref. [10] for ventricular fibrillation prediction and detection, demonstrating a strong capability to capture variations in heart rhythm.

Time-domain features include both simple indicators and measures derived from the signal dynamics, such as

- amplitude range—AR,
- peak-to-peak amplitude—PPA,
- mean amplitude—MA,
- median stepping increment—MSI,
- signal integral—SignInt,
- two variants of the root mean square (RMS) value, specific two ventricular fibrillation signals (RMS1 and RMS2) [18],
- mean slope—MS,
- median slope—MdS [19],
- a smoothed nonlinear energy operator—SNEO [20,21].

*Spectral features* were obtained by applying the Fast Fourier Transform (FFT) using 2048 points and a Hamming window over the analysis interval. These features include

- amplitude spectrum area—AMSA,
- centroid frequency—CF,
- peak frequency—PF,
- total energy—ENRG,
- spectral flatness measure—SFM,
- centroid power—CP,
- maximum power—MP,
- power spectrum analysis—PSA [19].

To assess signal complexity, we also included measures based on nonlinear dynamics. Among these are

- Hurst exponent (Hu) [22],
- scaling exponent—ScE,
- logarithm of absolute correlations—LAC [23],
- the median increment derived from the Poincaré plot (MSI) [24].

In addition, two entropy-based metrics were calculated:

- entropy—WE [25],
- spectral entropy—SpeEnt [26].

**Figure 4:**
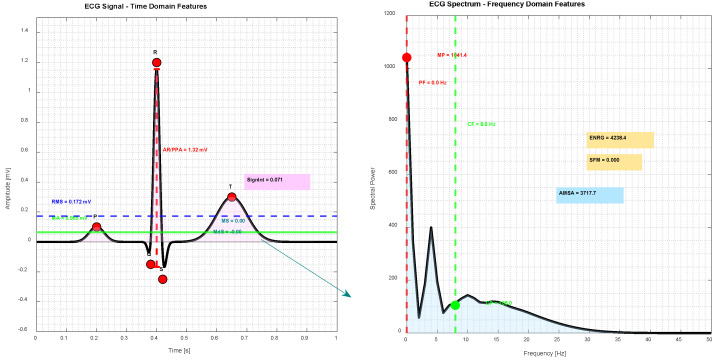
Time domain and frequency domain features on ECG signal.

Figure 4 exemplifies the time domain (RMS, MA, AR/PPA, MS, MdS, SignInt) and frequency domain features (PF, MP, CF, AMSA, SFM, ENRG) on a stylized ECG signal.

We used all features mentioned in this section as part of the extended set of features based on which we obtained the results of this paper.

We exclusively used lead II for extracting the ECG features required to classify certain supraventricular arrhythmias and sinus rhythms. The choice of this lead is based on both clinical and practical considerations; however, we acknowledge that this approach also has limitations, as we discuss in Appendix A.

This study deliberately explores a practical scenario in which only a single lead (lead II) is available, in order to simulate real-world situations in mobile applications, telemedicine, or automated screening. This approach may be valuable in resource-limited systems, in regions without access to a full 12-lead ECG, or in preliminary triage stages.

## 4. Results

The implementation phase followed two main directions, namely grouping the features into two major categories. The first category includes features related to waveform morphology and occurrence, presented in Section 3.5.1, while the second category comprises features proposed by us, pertaining to the time domain, spatial domain, and signal-complexity measures, presented in Section 3.5.2. Additionally, we considered general subject-specific features such as age, sex, ventricular rate, and atrial rate, which were taken into account regardless of feature grouping.

Regarding classification, we performed classification of the data into all 11 classes, noting that 3 classes—AVNRT, SAAWR, and AVRT—are very poorly represented. Due to this limitation and because these 11 pathology classes belong to 4 major groups, we also conducted classification by grouping the 11 categories into 4 groups (see Table 1). This grouping has been adopted in other studies as well, and besides the medical rationale related to their belonging to main classes, it facilitates comparison of our results with those reported in other works using the same database.

**Figure 5:**
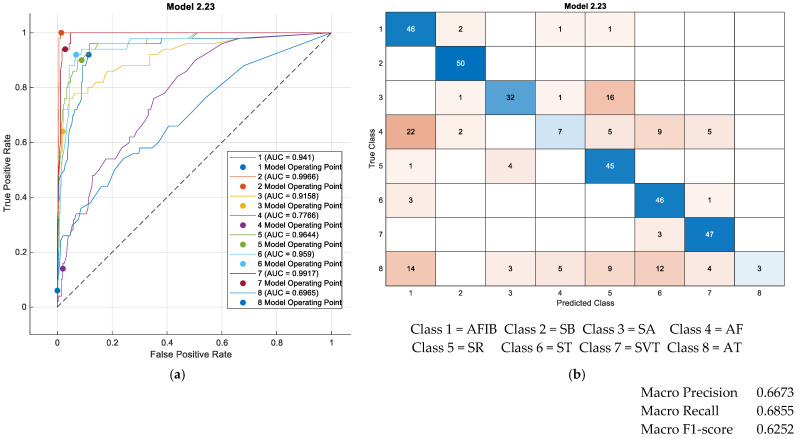
Classification results for the best classifier (**classification rate = 69%** by Ensemble Bagged Trees); classification of the eight classes with **29 features** for testing, with 50 examples from each class (wave form features and all temporal features (our features), all spectral features (our features), and nonlinearity measures (our features)). (**a**) Test ROC curve for eight classes and 50 examples for every class. (**b**) Test confusion matrix for eight classes and 50 examples for every class.

For the classification task involving eight classes (excluding the sparsely represented classes AVNRT, SAAWR, and AVRT), a subset of 50 randomly selected examples per class was reserved for testing, while the remaining samples were employed for training classifiers available within the MATLAB 2023b Classification Learner app. The results obtained using the Ensemble Bagged Trees classifier, which achieved the highest classification accuracy, are presented in Figure 5.

The confusion matrix shown in Figure 5 (right) reveals that the ST (Sinus Tachycardia) and AT (Atrial Tachycardia) classes could not be reliably distinguished from the other classes. The poor discrimination of the AT class (see Figure 5a,b) may be attributed to the relatively small sample size of this class (121 instances) compared to the others. Furthermore, even when employing a neural network trained with consideration of class imbalance, satisfactory performance for this class was not attained. The ROC curves and corresponding AUC indices depicted in Figure 5 demonstrate that certain classes (classes 2, 5, 6, and 7) were well-differentiated.

The classifiers were used with the default settings provided by MATLAB R2023b, as the primary objective was to obtain an overall perspective on the classification performance of the dataset rather than to perform extensive hyperparameter tuning. The results can be reproduced using the Classification Learner app in MATLAB R2023b with default configuration.

**Figure 6:**
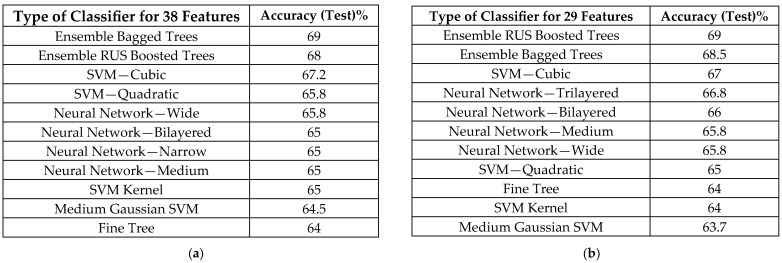
Comparative results for classification of the eight classes for testing, with 50 examples from each class. (**a**) Classification results for the best classifier (**classification rate= 69%** by Ensemble Bagged Trees); classification of the eight classes with **38 features** for testing, with 50 examples from each class (wave form features and all temporal features (our features), all spectral features (our features), and nonlinearity measures (our features)). (**b**) Classification results for the best classifier (**classification rate= 69%** by Ensemble Bagged Trees); classification of the eight classes with **29 features** for testing, with 50 examples from each class (only all temporal features (our features), all spectral features (our features), and nonlinearity measures (our features)).

Figure 6 presents classification outcomes obtained with additional classifiers available in the MATLAB Classification Learner app, evaluated for both the 38-feature (Figure 6a) and 29-feature subsets (Figure 6b).

Next, we present the results of classifying the data into the four major primary disease categories included in this database. It is important to note that for all classifications involving these four classes, the testing phase was conducted by reserving 500 examples per class. This approach results in more meaningful overall classification accuracy compared to evaluations based on testing 15–20% of the entire dataset. When a fixed proportion of data is allocated for testing, there are two possible scenarios: either the test samples are randomly selected from the entire dataset or the fixed proportion is applied within each class individually. In the case of highly imbalanced datasets, neither approach yields an accurate or fair classification accuracy. It is considerably more appropriate to test using the same number of examples per class. This strategy prevents an artificial inflation of the overall accuracy that can occur when testing involves a larger number of examples from a dominant class that is easily distinguishable and can be readily learned by the classifier, without requiring it to effectively differentiate the other classes.

**Figure 7:**
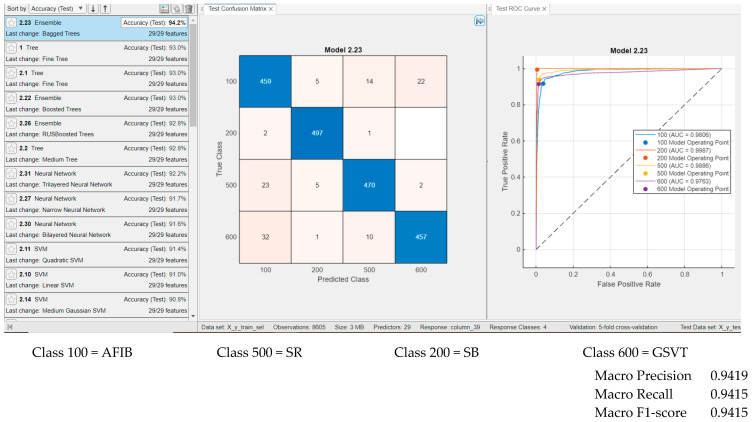
Results for classifiers with the best classification results (**classification rate = 94.2%** for Ensemble Bagged Trees) in four classes with **29 features** (age, gender, ventricular rate, atrial rate, and all temporal features (our features), all spectral features (our features), and nonlinearity measures (our features)), and for testing, with 500 examples from each class.

Figure 7 presents the classification results using temporal features, all spectral features, and nonlinear measures proposed by our approach. Figure 7 illustrates the performance of the Ensemble Bagged Trees classifier, which achieved the highest accuracy, 94.2%, when testing on 500 examples per class. The ROC curves and AUC values exceed 0.97 for each class, with a maximum AUC of 0.9989 observed for the Sinus Bradycardia group. Classification results for other classifiers are also shown, generally yielding performance comparable to that of Ensemble Bagged Trees. For instance, neural networks with the default settings provided by MATLAB deliver promising results, and classification accuracy could likely be improved further through parameter optimization.

**Figure 8:**
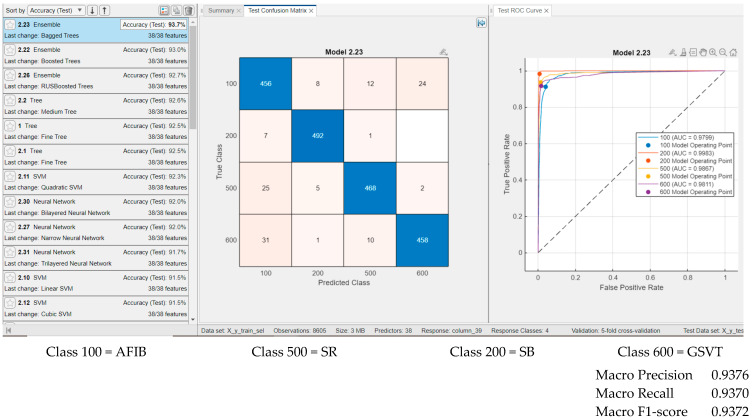
Results for classifiers with the best classification results (**classification rate = 93.7%** for Ensemble Bagged Trees) in four classes with **39 features** (wave form features and all temporal features (our features), all spectral features (our features), and nonlinearity measures (our features)), and for testing, with 500 examples from each class.

Figure 8 presents the classification results for the groups using general ECG waveform features, temporal features, spectral features, and nonlinear signal measures—that is, all features described in Section 3.5. Testing was conducted on 500 examples per group. The highest classification accuracy was achieved with the Ensemble Bagged Trees classifier at 93.7%, although other classifiers produced comparable results. As observed in Figure 8, all four classes exhibit similarly high classification rates, indicating that no class is indistinguishable from the others. The ROC curves and corresponding AUC indices exceed 0.97 for all four classes.

**Figure 9:**
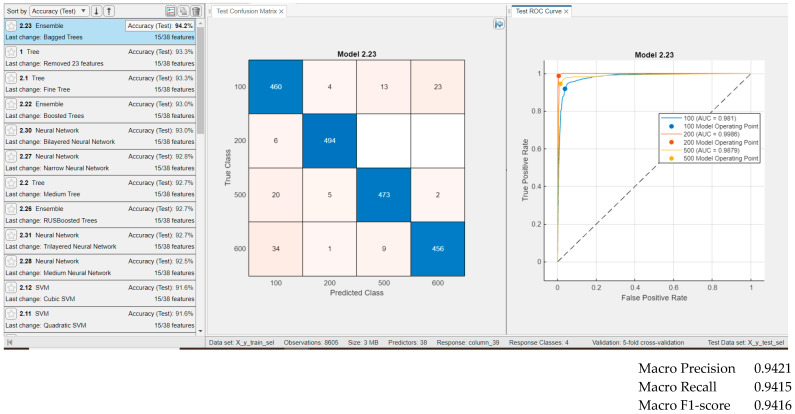
Results for classifiers with the best classification results in four classes with **15 features** (feature selection MRMR) and for testing, with 500 examples from each class (**Classification Rate: 94.2%**).

The results obtained with other classifiers using the same features and 500 examples per class are presented in Figure 9. It can be observed that both ensemble classifiers and neural networks achieve very good and comparable performance.

**Figure 10:**
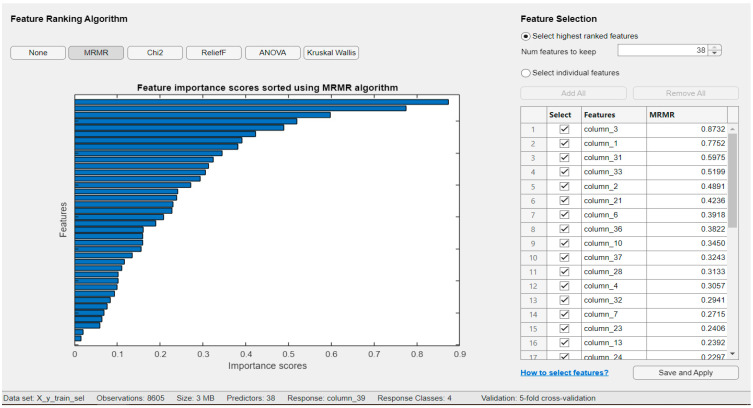
Feature importance score sorted using MRMR algorithm. (In descendent order, from column 3 to column 24, feature selected is ventricular rate, age, SFM = spectral flatness measure, FuzzyEn = Fuzzy Entropy, gender, SNEO = Smoothed Nonlinear Energy Operator, QTInterval, Hurst Exponent, QRS count, centroid power, approximate Entropy, SpeEnt = Spectral Entropy, atrial rate, QT Corrected, MSI = median stepping increment).

We also evaluated the case where only a subset of features was retained by applying feature selection using the Minimum Redundancy Maximum Relevance (MRMR) algorithm available in the MATLAB Classification Learner app. The classification results for the four classes using the top 15 most important features are presented in Figure 9. Testing was performed with 500 examples per class. The feature importance scores calculated by the MRMR algorithm are shown in Figure 10, where the score for each individual feature is also indicated. The top 15 features selected by the MRMR algorithm are as follows:

- ventricular rate,
- age,
- SFM = spectral flatness measure,
- FuzzyEn = Fuzzy Entropy,
- gender,
- SNEO = Smoothed Nonlinear Energy Operator,
- QTInterval,
- Hurst Exponent,
- QRS count,
- centroid power,
- approximate Entropy,
- SpeEnt = Spectral Entropy,
- atrial rate,
- QT Corrected,
- MSI = median stepping increment.

It is observed that, in general, the selected features predominantly consist of spectral features and signal nonlinearity measures, as well as ventricular rate, age, and gender.

**Figure 11:**
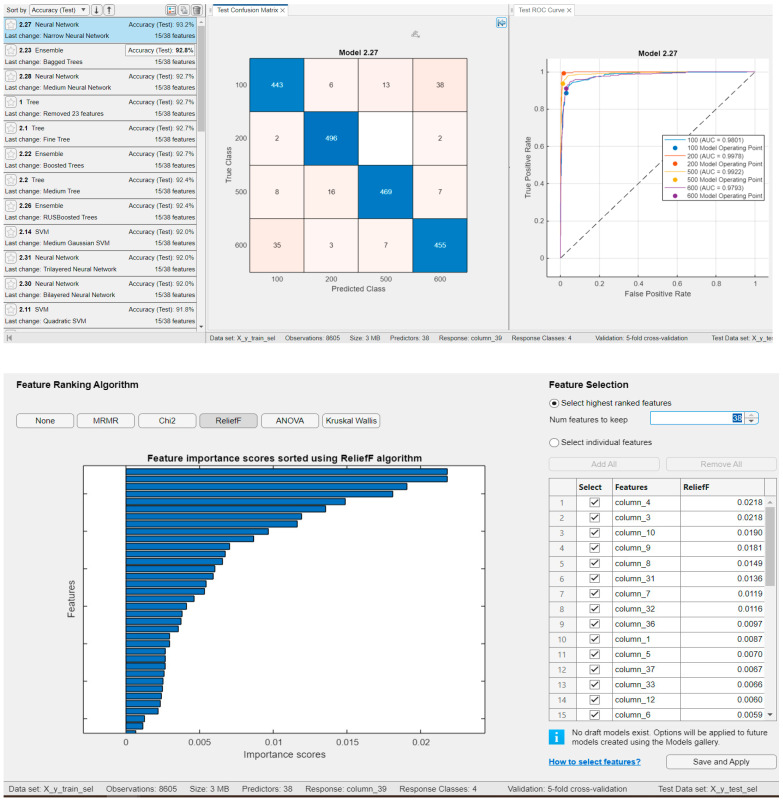
Results for classifiers with the best classification results in four classes with **15 features** (ReliefF algorithm selection) and for testing, with 500 examples from each class (**Classification Rate: 93.2%**); Feature importance score using ReliefF algorithm, test confusion matrix, and test ROC curve are presented for the best classifier, i.e., for Narrow Neural Network.

Other feature selection methods available in MATLAB^®^ were also evaluated; however, the results demonstrated that the best performance was achieved using the MRMR algorithm. Nevertheless, these alternative results are presented in the following figure. Specifically, Figure 11 shows the classification outcomes using the ReliefF feature selection method, where the top 15 features were retained, yielding a classification accuracy of 93.2% with Narrow Neural Networks.

**Figure 12:**
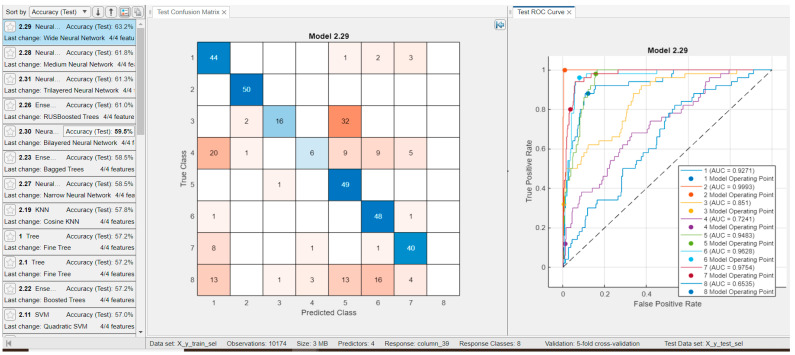
Results for classifiers with the best classification results in eight classes with only **four features** (age, gender, ventricular rate, atrial rate) and for testing, with 50 examples from each class (**Classification Rate: 63.2%**); Test confusion matrix and test ROC curve are presented for the best classifier, i.e., for Wide Neural Network.

**Figure 13:**
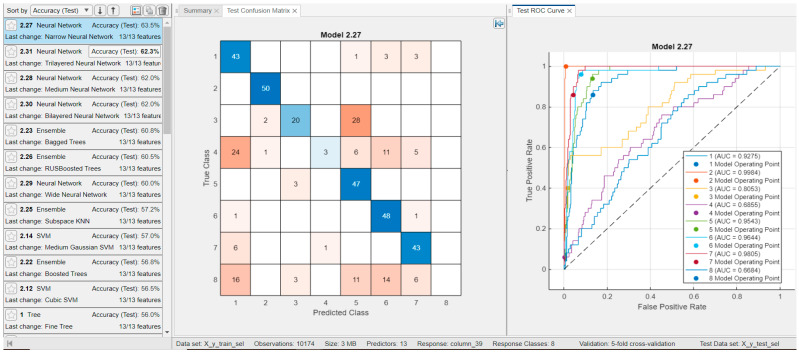
Results for classifiers with the best classification results in eight classes with only **13 features** (Features extracted from lead II [11] ventricular rate in beats per minute (BPM), atrial rate, QRS duration, QT interval, R axis, T axis, QRS count, Q onset, Q offset, mean of RR interval, variance of RR interval, RR interval count) and for testing, with 50 examples from each class (**Classification Rate: 63.5%**); Test confusion matrix and test ROC curve are presented for the best classifier, i.e., for Narrow Neural Network.

We evaluated the relevance and discriminative power of general features—such as age, gender, ventricular rate, and atrial rate—by performing classification into the eight pathological classes based solely on these variables. Additionally, we assessed the informativeness of the features proposed in ref. [11], extracted from a single lead, by conducting a similar classification into the same eight classes. The results, illustrated in Figure 12 and Figure 13 respectively, show that using only four general features, a classification accuracy of 63.2% was achieved with a neural network model. In comparison, using 13 features extracted from lead DII yielded a slightly higher accuracy of 63.5%.

## 5. Discussion

Signal classification methods have undergone remarkable evolution in recent years, both in terms of classification results and in terms of applied signal analysis. Thus, deep learning-based methods are the latest trend when it comes to signal classification. These techniques range from applying 1D or 2D signals to deep learning neural networks to techniques for extracting defining signal features. Thus, for heartbeat classification, studies in recent years have reported an evolution of multiclass results, and in terms of innovative technical aspects, the elimination of the dependence on R-peaks, which is revolutionary for real-time applications, end-to-end processing, i.e., from raw signal to classification without manual intervention, and adaptability to noise by integrating Kalman-type adaptive filters modified in the architecture, should be mentioned.

The paper [27] uses the Stockwell transform for time–frequency conversion, eliminating the need for R-peak detection. It combines CNN with Transformer self-attention mechanisms to capture long-term dependencies. It achieves 99.58% accuracy on MIT-BIH and 97.8% on Icentia11k.

In ref. [28], a modified Kalman filter for pre-processing and self-attention mechanisms for automatic feature extraction is used. It demonstrates exceptional robustness to noise: SNRimp 24.00 dB and RMSE 0.055 for SNR −5dB.

The work [29] implements 24 densely connected CNN layers for hierarchical feature extraction, combined with BiLSTM for bidirectional temporal modeling. It achieves >99% accuracy on MIT-BIH for multiple arrhythmia classes.

The paper [30] converts ECG signals into RGB spectrograms using continuous wavelet transform (CWT), applying transfer learning with CNNs pre-trained on ImageNet. It achieves >95% accuracy for arrhythmia classification.

Ref. [31] combines 2DCNN for spatial features with GRU for temporal modeling, focusing on “shockable” arrhythmias. It achieves >94% accuracy for rapid detection of life-threatening arrhythmias.

If we extrapolate the results obtained for similar tasks on other databases, we can assume that more sophisticated deep learning methods like a hybrid CNN transformer using Stockwell transform [27] or self attention-based auto encoders [28] or deep CNN and BiLSTM [29] could improve the accuracy of the method, but there will be a need to assess the technical feasibility due to the need of huge datasets and the time complexity of those approaches versus our current proposal. This evaluation of more sophisticated deep learning approaches will be a focus of further work for our team.

The analysis conducted on this dataset and on its potential for classification into eight or four classes has led to several conclusions. Firstly, many features do not contribute relevant information for the classification task. This is supported by the results obtained using the MRMR feature selection algorithm: by retaining only the top 15 most representative features, similar or slightly improved classification performance was achieved compared to the scenarios using 29 or even 39 features.

In the scenario of classification into eight classes using only demographic and basic rhythm-related information (age, gender, ventricular rate, and atrial rate), a classification accuracy of 63.2% was obtained. This indicates that these features carry significant discriminative value and can serve as a strong baseline for initial differentiation between classes. Furthermore, when additional features related to the QRST waves and their intervals were included—such as QRS duration, QT interval, corrected QT interval, R axis, T axis, QRS count, Q onset, Q offset, and T offset (Figure 9)—the classification accuracy remained unchanged at 63.2%. This suggests that, in some cases, including more features derived from multiple ECG channels and wave segments does not necessarily enhance performance and may even burden the classifier, leading to lower accuracy and increased computational costs.

Because the database is relatively new, established in 2022, the number of papers that have reported results on this database is relatively small, and a comparative analysis requires the use of the same database.

However, there are some works that use this database for the results obtained by 2D processing using deep learning neural networks, namely convolutional neural networks (CNN) [32,33]. In the paper [34], a comparative analysis of the classification performances of three distinct types of spatial representations of ECG arrhythmia signals, when combined with various convolutional neural network architectures, is presented, the reported results being presented in Table 2.

Table 2 summarizes the classification results. It can be observed that the features proposed in this study yield comparable accuracy to those reported in ref. [11]. In addition to temporal features, spectral features, and nonlinearity measures, several frequency- and waveform-related features were identified as relevant, such as ventricular rate (BPM), atrial rate (BPM), QRS duration, QT interval, and corrected QT interval.

The difference of less than 2% compared to the results reported in ref. [11] may be attributed to the use of different test sets. However, in multiclass imbalanced data scenarios, using an equal number of test examples per class provides a more reliable evaluation than percentage-based sampling. Additionally, the number of runs used to report average performance has a considerable impact. Taking these factors into account, we argue that our results are comparable in classification performance to those in ref. [11], with the added advantage of a relatively small number of extracted features and the use of a single ECG channel, as well as obtaining a significantly smaller run time of the used algorithms. Specifically, in our proposed approach, only lead DII was processed.

**Figure 14:**
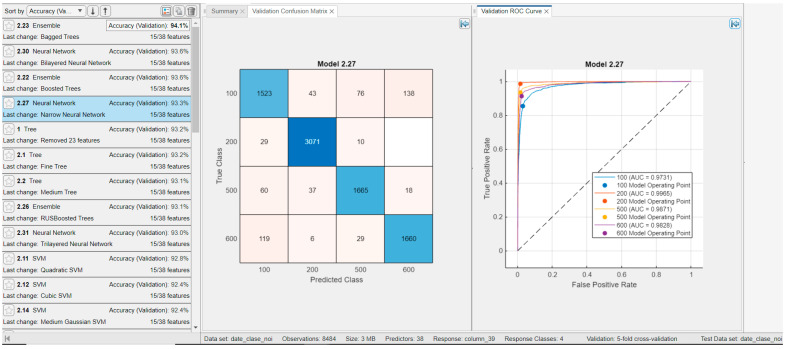
Results for classifiers achieving the best performance in four-class classification using 15 features selected by the ReliefF algorithm and evaluated on a test set representing 20% of the data (80:20 train–test split). The classification rate is 93.3%. Feature importance scores from ReliefF, the validation confusion matrix, and the validation ROC curve are shown for the best-performing model, the Narrow Neural Network.

**Figure 15:**
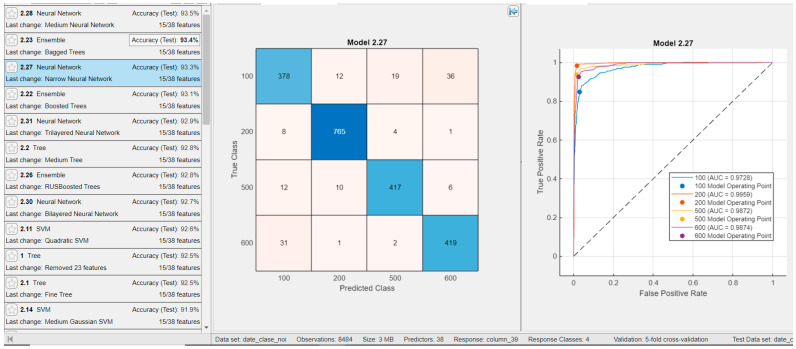
Results for classifiers achieving the best performance in four-class classification using 15 features selected by the ReliefF algorithm, evaluated on a test set representing 20% of the data (80:20 train–test split). The classification rate is 93.3%. Feature importance scores from ReliefF, the test confusion matrix, and the test ROC curve are shown for the best-performing model, the Narrow Neural Network.

Figure 14 and Figure 15 present the results of splitting the data into training and testing sets with an 80:20 ratio (as described in paper [11]), showing the confusion matrix and ROC curve for the validation set and, respectively, the test set for the Narrow Neural Network, which achieved a performance of 93.3% on the test set. The validation and test sets yielded similar results, indicating that the model is not overfitting and demonstrates good generalization capability.

## 6. Conclusions

The results demonstrate that a limited set of general features (age, sex, ventricular and atrial rate), combined with spectral domain features and nonlinear signal measures, can provide highly relevant information for ECG signal classification. In the eight-class classification scenario, these basic features alone achieved an accuracy of 63.2%, comparable to that obtained using a set of 13 morphological features from the literature. Expanding the feature set to 29 or 39—including temporal features—led to an improved classification accuracy of up to 69%.

For four-class classification, using only 15 features selected through MRMR resulted in an accuracy of 94.2%, showing that an efficient and well-targeted feature selection process can significantly reduce model complexity without compromising performance. In comparison, other methods from the literature require 230 features extracted from all 12 ECG leads to achieve 96% accuracy, but with substantially higher computational cost.

The main advantage of the proposed method lies in its processing efficiency: all features are extracted from a single ECG lead (DII), significantly reducing preprocessing, data storage, and classification time—by an estimated factor of about 12 compared to approaches using all 12 leads. Therefore, our solution delivers competitive performance while being simpler to implement in clinical applications or automated diagnostic systems.

As further work, we intend to evaluate more sophisticated deep learning methods for arrythmia classification and to compare them with our current approach.

## Figures and Tables

**Table 1:** Rhythm group and rhythm information.

Rhythm Group	Rhythm Information	Rhythm Name
AFIB	AFIB = 1780	Atrial Fibrillation
AFIB	AF = 445	Atrial Flutter
SB	SB = 3888	Sinus Bradycardia
SR	SA = 399	Sinus Arrythmias
SR	SR = 1826	Sinus Rhythm
GSVT	ST = 1568	Sinus Tachycardia
GSVT	SVT = 587	Supraventricular Tachycardia
GSVT	AT = 121	Atrial Tachycardia
GSVT	AVNRT = 16	Atrioventricular Node Reentrant Tachycardia
GSVT	SAAWR = 7	Sinus Atrium to Atrial Wandering Rhythm
GSVT	AVRT = 8	Atrioventricular Reentrant Tachycardia

**Table 2:** Summary of the classification results obtained using a balanced dataset with an equal number of examples per class (i.e., 50 samples per class for the eight-class classification task, and 500 samples for the four-class scenario).

Paper	No Classes and Info About the Test Dataset	No Features	Classif. Rate	Measure Performance	Features
our	eight classes 50 sample tests for each class	4 features	63.2%		age, gender, ventricular rate, atrial rate
[11]	eight classes 50 sample tests for each class	13 features	63.5%		**Features extracted from lead II [11]** ventricular rate in beats per minute (BPM), atrial rate, QRS duration, QT interval, R axis, T axis, QRS count, Q onset, Q offset, mean of RR interval, variance of RR interval, RR interval count
our	eight classes 50 sample tests for each class	29 features	69%	Macro Precision 0.6673 Macro Recall 0.6855 Macro F1-score 0.6252	age, gender, ventricular rate, atrial rate + temporal features (our features), spectral features (our features), and nonlinearity measures (our features)
our	eight classes 50 sample tests for each class	39 features	69%	Macro Precision 0.6677 Macro Recall 0.6850 Macro F1-score 0.6253	wave form features [11] and temporal features (our features), spectral features (our features), and nonlinearity measures (our features)
our	four classes 500 sample tests for each class	29 features	94.2%	Macro Precision 0.9419 Macro Recall 0.9415 Macro F1-score 0.9415	age, gender, ventricular rate, atrial rate + temporal features (our features), spectral features (our features), and nonlinearity measures (our features)
our	four classes 500 sample tests for each class	39 features	93.7%	Macro Precision 0.9376 Macro Recall 0.9370 Macro F1-score 0.9372	wave form features [11] and temporal features (our features), spectral features (our features), and nonlinearity measures (our features)
our	four classes 500 sample tests for each class	15 features selection MRMR	94.2%	Macro Precision 0.9421 Macro Recall 0.9415 Macro F1-score 0.9416	ventricular rate, age, SFM = spectral flatness measure, FuzzyEn = Fuzzy Entropy, gender, SNEO = Smoothed Nonlinear Energy Operator, QTInterval, Hurst Exponent, QRS count, centroid power, approximate Entropy, SpeEnt = Spectral Entropy, atrial rate, QT Corrected, MSI = median stepping increment
our	four classes Train set:Test set = 80:20 (as in the paper [11])	15 features selection MRMR	93.3%	Macro Precision 0.9421 Macro Recall 0.9415 Macro F1-score 0.9416	ventricular rate, age, SFM = spectral flatness measure, FuzzyEn = Fuzzy Entropy, gender, SNEO = Smoothed Nonlinear Energy Operator, QTInterval, Hurst Exponent, QRS count, centroid power, approximate Entropy, SpeEnt = Spectral Entropy, atrial rate, QT Corrected, MSI = median stepping increment
[11]	four classes Train set:Test set = 80:20	230 features	96% calculated by us from the information provided in the paper [11] by Macro Precision 0.966 Macro Recall 0.964 Macro F1-score 0.965	Macro Precision 0.966 Macro Recall 0.964 Macro F1-score 0.965	**Features extracted from lead II include:** ventricular rate in beats per minute (BPM), atrial rate, QRS duration, QT interval, R axis, T axis, QRS count, Q onset, Q offset, mean of RR interval, variance of RR interval, RR interval count **Features extracted from 12 leads contain:** mean and variance of height, width, prominence for QRS complex, non-QRS complex, and valleys
[34]	four classes		89.3%	AUC 98.2 (±0.2) Sensitivity 73 (±2.2) Specificity 98.1 (±1.7) Precision 77.2 (±5.3) F1 72.4 (±2.6) Kappa 56.4 (±3.9)	CUSTOM CNN MODEL ARCHITECTURE Dataset of 128 x 384 matrix containing ECG data for each patient

## Data Availability

The data presented in this study are openly available in [physionet] at [https://physionet.org/content/ecg-arrhythmia/1.0.0/] (accessed on 1 May 2025) and [https://springernature.figshare.com/articles/dataset/Metadata_record_for_A_12-lead_electrocardiogram_database_for_arrhythmia_research_covering_more_than_10_000_patients/11698521] (accessed on 1 May 2025).

## Appendix A

Lead II is one of the most commonly used leads in clinical practice for heart rhythm analysis, especially in continuous monitoring (Holter, portable monitors, wearable devices), for the following reasons:

▪ It has an electrical axis approximately parallel to the normal conduction of the impulse through the atria and ventricles.
▪ It allows clear visualization of the P wave, PR interval, and QRS complex, which are essential for the analysis of supraventricular arrhythmias.
▪ It is frequently used in the context of rapid clinical rhythm assessment, often being the only lead available in portable or non-invasive settings.

Nevertheless, there are important limitations that must be acknowledged:

▪ In atrial fibrillation with fine f waves, atrial activity may be difficult to detect in lead II and is more clearly seen in V1.
▪ In atrial flutter, the characteristic “sawtooth” pattern may be masked in lead II, especially in atypical cases; V1 and aVL may be more informative.
▪ Supraventricular tachycardias (AVNRT, AVRT) may present retrograde P waves, which are either absent or superimposed on the QRS complex in lead II, requiring visualization in aVR or V1 for clarification.
▪ Migratory atrial rhythms or rhythms with an ectopic focus (e.g., sinus atrium to atrial wandering rhythm) involve subtle differences in P-wave morphology, which can be difficult to distinguish in lead II, especially if the voltage is low.

These limitations can lead to classification errors in cases where P-wave morphology is essential for discrimination between similar classes.

The proposed model is not a substitute for a complete 12-lead ECG diagnosis; rather, it can serve as an automated pre-triage or alerting tool within broader clinical workflows. We anticipate that extending the method by integrating additional leads (e.g., V1, aVR) could increase sensitivity and specificity for arrhythmias in which P-wave morphology is ambiguous or atrial activity is poorly represented in lead II.

**Table A1:** Summary of arrhythmia class and clinical observations and limitations in lead II.

Arrhythmia Class	Visibility in Lead II	Clinical Observations and Limitations	Recommended Optimal Lead(s)	No. of Examples in Database
Sinus Rhythm	Good	P waves and QRS are clearly visible in lead II.	II	1826
Sinus Tachycardia	Good	Easily identifiable by increased heart rate and normal P–QRS morphology.	II	1568
Sinus Bradycardia	Good	Low heart rate, but P waves and QRS remain clear.	II	3888
Sinus Arrhythmia	Good	Cyclical variations in RR interval observed; visible in lead II.	II	399
Atrial Fibrillation	Variable	F waves may be fine and hard to distinguish; V1 may be more informative.	V1	1780
Atrial Flutter	Limited	F waves may be masked in lead II; V1 or aVL better highlight the “sawtooth” pattern.	V1, aVL	445
Atrial Tachycardia	Acceptable	P-wave morphology may be altered but is identifiable in lead II in many cases.	V1, aVR, aVL	121
Supraventricular Tachycardia (SVT)	Partial	P waves may be absent or retrograde and superimposed on QRS; aVR and V1 may be more useful.	aVR, V1	587
AVNRT (AV Node Reentrant Tachycardia)	Limited	Retrograde P waves often invisible in lead II; can be highlighted in aVR/V1.	V1, aVR	16
AVRT (Atrioventricular Reentrant Tachycardia)	Limited	Atrial activity may be hidden in lead II; other leads can clarify the P–QRS relationship.	V1, aVR, III	8
Sinus Atrium to Atrial Wandering Rhythm	Variable	Fine changes in P waves may be hard to distinguish in lead II; other leads (e.g., V1) are useful.	V1, aVL, III	7
